# Supplementary material for: Machine learning-based on model for explain risk of 24-hour death in critically ill patients in the prehospital setting: A retrospective cohort study
Source: PLoS One. 2026 Feb 12;21(2):e0341860. doi: 10.1371/journal.pone.0341860 (PMC12900353; doi:10.1371/journal.pone.0341860)
Supplement: S1 Table — (DOCX) [file pone.0341860.s003.docx]

**S1 Table** The predictive ability of single parameter for mortality risk in critically ill patients

| Variable | Feature groups | AUC | SE | 95% CI |
| --- | --- | --- | --- | --- |
| Admission_blood_oxygen | 4 | 0.858 | 0.0332 | 0.829 to 0.885 |
| Admission_DBP | 4 | 0.841 | 0.0344 | 0.810 to 0.869 |
| Admission_SBP | 4 | 0.840 | 0.0367 | 0.809 to 0.868 |
| Prehospital_SBP | 4 | 0.835 | 0.0368 | 0.803 to 0.863 |
| Prehospital_DBP | 9 | 0.811 | 0.040 | 0.778 to 0.841 |
| Prehospital_blood_oxygen | 9 | 0.805 | 0.0419 | 0.772 to 0.835 |
| Awareness | 9 | 0.784 | 0.0316 | 0.750 to 0.816 |
| Admission_respiratory_frequency | 9 | 0.748 | 0.0562 | 0.712 to 0.781 |
| Prehospital_heart_rate | 9 | 0.726 | 0.0549 | 0.689 to 0.760 |
| BMV | 14 | 0.723 | 0.0506 | 0.686 to 0.757 |
| PRL | 14 | 0.719 | 0.0513 | 0.682 to 0.754 |
| Admission_heart_rate | 14 | 0.718 | 0.0549 | 0.681 to 0.753 |
| CPR | 14 | 0.713 | 0.0519 | 0.676 to 0.748 |
| Prehospital_respiratory_frequency | 14 | 0.686 | 0.0572 | 0.648 to 0.722 |
| PER | 19 | 0.683 | 0.0516 | 0.645 to 0.720 |
| Admission_shock_index | 19 | 0.654 | 0.0359 | 0.615 to 0.691 |
| Prehospital_shock_index | 19 | 0.642 | 0.0381 | 0.603 to 0.680 |
| Admission_temperature | 19 | 0.617 | 0.0624 | 0.577 to 0.655 |
| Prehospital_temperature | 19 | 0.604 | 0.0536 | 0.564 to 0.643 |
| Transit_time | 24 | 0.581 | 0.0467 | 0.541 to 0.620 |
| Age | 24 | 0.568 | 0.0438 | 0.528 to 0.608 |
| Oxygen_inhalation | 24 | 0.566 | 0.0473 | 0.527 to 0.606 |
| Lung_diseases | 24 | 0.548 | 0.0437 | 0.508 to 0.587 |
| Hypertension | 24 | 0.539 | 0.0465 | 0.499 to 0.578 |
| Liver_kidney_diseases | 29 | 0.523 | 0.0455 | 0.483 to 0.562 |
| gender | 29 | 0.512 | 0.0475 | 0.472 to 0.552 |
| Diabetes | 29 | 0.510 | 0.0477 | 0.470 to 0.550 |
| Stroke | 29 | 0.507 | 0.0476 | 0.467 to 0.547 |
| CHD | 29 | 0.506 | 0.047 | 0.466 to 0.546 |

BMV: Bag-mask ventilation; CPR: cardio-pulmonary resuscitation; CHD: coronary heart disease; CI: confidence interval; DBP: diastolic blood pressure; PER: pupils equal and round; PRL: pupils reactive to light; SBP: Systolic blood pressure; SE: standard error;
