# Supplementary material for: Machine learning-based on model for explain risk of 24-hour death in critically ill patients in the prehospital setting: A retrospective cohort study
Source: PLoS One. 2026 Feb 12;21(2):e0341860. doi: 10.1371/journal.pone.0341860 (PMC12900353; doi:10.1371/journal.pone.0341860)
Supplement: S2 Table — Performance of the top five ML models for mortality risk prediction based on nine features. (DOCX) [file pone.0341860.s004.docx]

**S2 Table** Performance of the top five ML models for mortality risk prediction based on nine features

| Dataset | Model | AUC | Sensitivity | Specificity | PPV | NPV | Accuracy | F1 score |
| --- | --- | --- | --- | --- | --- | --- | --- | --- |
| Training | SVM | 0.889 | 0.897 | 0.775 | 0.983 | 0.341 | 0.889 | 0.938 |
|  | MLP | 0.960 | 0.643 | 0.850 | 0.989 | 0.507 | 0.938 | 0.966 |
|  | RF | 0.985 | 0.961 | 1.000 | 1.000 | 0.635 | 0.963 | 0.980 |
|  | KNN | 0.955 | 0.812 | 1.000 | 1.000 | 0.267 | 0.824 | 0.896 |
|  | LightGBM | 0.936 | 0.844 | 0.925 | 0.994 | 0.289 | 0.849 | 0.913 |
| Testing | SVM | 0.908 | 0.935 | 0.636 | 0.966 | 0.467 | 0.910 | 0.950 |
|  | MLP | 0.805 | 0.939 | 0.591 | 0.962 | 0.464 | 0.910 | 0.951 |
|  | RF | 0.863 | 0.943 | 0.636 | 0.967 | 0.500 | 0.918 | 0.955 |
|  | KNN | 0.837 | 0.776 | 0.773 | 0.974 | 0.236 | 0.776 | 0.864 |
|  | LightGBM | 0.891 | 0.854 | 0.682 | 0.968 | 0.294 | 0.840 | 0.907 |

AUC: area under the curve; KNN: K-nearest neighbor; LightGBM: light gradient boosting machine; NPV: negative; PPV: positive predictive value; predictive value; ML: machine learning; MLP: Multilayer perception; RF: random forest; SVM: support vector machine;
