# Supplementary figures and images for: Machine learning-based on model for explain risk of 24-hour death in critically ill patients in the prehospital setting: A retrospective cohort study
Source: PLoS One. 2026 Feb 12;21(2):e0341860. doi: 10.1371/journal.pone.0341860 (PMC12900353; doi:10.1371/journal.pone.0341860)

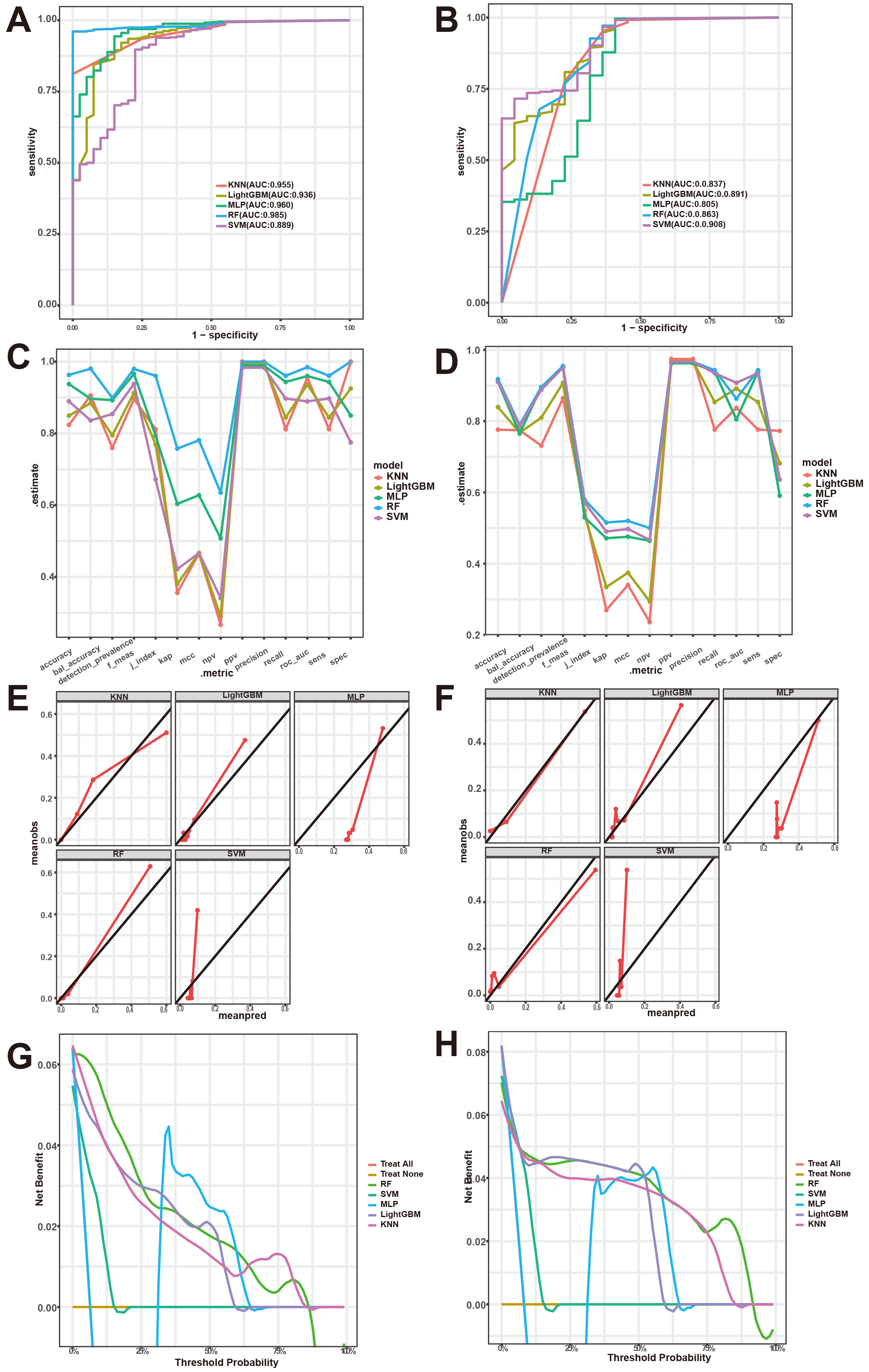

Supplement: S1 Fig — A and B: The receiver operating characteristics curve with AUCs; C and D: Parallel line graph of the evaluation metrics for the top five models. E and F: calibration curve analysis; G and H: Decision curves analysis of the top five models. (TIF) [file pone.0341860.s005.tif]
